# Lessons learned from automated screening of COVID-19 preprints: Tracey Weissgerber

**DOI:** 10.1093/eurpub/ckac129.127

**Published:** 2022-10-25

**Authors:** T Weissgerber, N Riedel, H Kilicoglu, C Labbe, P Eckmann, G ter Riet, J Byrne, G Cabanac, A Capes-Davis, A Bandrowski

**Affiliations:** 1 QUEST Center, BIH, Charité – Universitätsmedizin Berlin, Berlin, Germany; 2 School of Information Sciences, University of Illinois - Urbana-Champaign, Champaign, USA; 3 University Grenoble Alpes, CNRS, Grenoble, France; 4 Department of Neuroscience, University of California San Diego, San Diego, USA; 5 SciCrunch Inc., San Diego, USA; 6 Department of Cardiology, Amsterdam UMC, University of Amsterdam, Amsterdam, Netherlands; 7 Faculty of Medicine and Health, University of Sydney, Sydney, Australia; 8 UMR 5505 IRIT, Universite de Toulouse, Toulouse, France; 9 CellBank Australia, Children’s Medical Research Institute, Sydney, Australia

## Abstract

Preprints occupied the spotlight early in the pandemic, as scientists, the media and the public sought information on the evolving pandemic. While some in the scientific community embraced this shift, others were concerned about the quality of these papers, which had not yet undergone peer review. Furthermore, the flood of COVID-19 preprints quickly overwhelmed the scientific community's ability to monitor and assess new preprints. Automated screening tools that detect beneficial practices, or common problems, in preprints are one potential solution to this problem. These tools could potentially provide individualized feedback, allowing authors to improve their manuscripts prior to publication in a peer-reviewed journal. We have combined many tools into a single pipeline, called ScreenIT. ScreenIT assess factors such as open data and open code, blinding, randomization, power calculations, limitations sections, and data visualization problems. Since June 2020, we have used ScreenIT to screen and post daily reports on more than 23,000 new COVID-19 preprints deposited on bioRxiv and medRxiv. Results show that practices such as sharing data and code are relatively uncommon. Sample size calculations, blinding and randomization are rarely reported and most papers do not report the sex of participants, animals or samples. This work demonstrates the feasibility of using automated tools to rapidly screen many preprints in real time, and provide authors and readers with rapid feedback. However, this approach has important limitations. Automated screening tools can make mistakes. Tools can't always determine whether an item is relevant to a particular manuscript. Further studies are needed to determine whether feedback from automated tools is effective in encouraging authors to improve reporting.

